# Fellow-Workers and Their Doings

**Published:** 1914-04-04

**Authors:** 


### ROUND THE WORLD.

### Fellow-Workers and their Doings.

[The Editor Invites Early Information and Contributions Marked "Fellow-Workers."]

Dr. Walter Ramsden, M.D. Oxon., who has just been appointed University Lecturer in Chemical Physiology at Oxford, is well known to students there as the senior demonstrator in the physiological laboratories there. Dr. Ramsden did most of his clinical work at "Guy's," qualifying in 1896, whilst among other distinctions he was awarded the Radcliffe Travelling Fellowship in 1893. Some of his most important work has been published in the Proceedings of the Royal Society.

It does not appear to be generally known that the moving spirit in the foundation of the interesting museum of medico-legal specimens at University College Hospital has been Dr. F. J. Poynton, the well-known member of the staff of that institution and of the Great Ormond Street Hospital for Sick Children. In earlier years Dr. Poynton was connected with St. Mary's Hospital, where, with Dr. Alexander Paine, he initiated those researches on the bacteriology of acute rheumatism which have occasioned so much interest not only in this country but abroad.

University theses are seldom of such interest as that recently presented for the Cambridge M.D. by Dr. Philip Bahr, the subject matter of which covers the clinical and bacteriological investigation of sprue, a malady that has only lately been recognised as being not uncommon in England. Dr. Bahr, who is a son-in-law of Sir Patrick Manson, has come to the conclusion that sprue is largely due to yeast organisms, and, moreover, that it is by no means unlikely that many cases of intractable colitis in adults and "summer diarrhœa" in children are of a similar nature. Before going to Ceylon to study sprue Dr. Bahr spent a year in Fiji investigating dysentery, and subsequently collaborated with Dr. Glanvill Corney, formerly chief medical officer in Fiji, in the publication of an important article on this subject.

Mr. J. P. Buckley, M.S., F.R.C.S., who has been appointed honorary assistant surgeon at the Manchester Royal Infirmary, had a most successful career as a student of Cambridge and Victoria Universities and at the London Hospital, having distinguished himself in physiology and obtained a first-class Natural Science Tripos in 1904. Mr. Buckley, who is also on the staff of the Salford Royal Hospital, has held the posts of honorary medical officer to the Central Branch Manchester Royal Infirmary and clinical assistant to the Queen's Hospital for Children, Hackney.

Mr. Joseph Cunning, who not very long ago succeeded to the charge of surgical in-patients at the Royal Free Hospital, has found it necessary to resign his post as surgeon to the Cancer Hospital on account of the demands which the Gray's Inn Road institution makes upon his time. Mr. Cunning is M.B., B.S. Melbourne, as well as F.R.C.S. England, and is, we believe, an Australian by birth. He is the author of "Aids to Surgery," which is extremely popular with medical students. He was elected direct to the senior staff of the Cancer Hospital a few years ago, without ever having passed through the usual probationary years as assistant surgeon; this in itself is a somewhat unusual distinction in London. Mrs. Cunning is also qualified to practise medicine and surgery.

Dr. Charles Eddowes, who has recently been presented with a substantial testimonial from old friends and patients, had, up to his retirement a few months ago, practised for over half a century at Shrewton, in Wiltshire, and his seventy-seventh birthday was made the occasion of the presentation. Dr. Eddowes is an old Westminster Hospital man and qualified as a Member of the Royal College of Surgeons as far back as 1861.

Dr. Elgar Down, the new honorary physician to the Royal Albert Hospital at Devonport, qualified from the London Hospital as an M.R.C.S., L.R.C.P. in 1895, ten years afterwards becoming an F.R.C.S. Edinburgh.

Mr. F. Richardson Cross, M.B., F.R.C.S., who is going to take the chair at the Ophthalmological Congress to be held during April in London, is president of the Ophthalmological Society, and very well known in the West of England. Formerly a student of King's College Hospital, Mr. Cross eventually settled in Bristol, where he has held many public appointments, and is at the present time consulting ophthalmic surgeon to the Royal Infirmary, surgeon to the Eye Hospital, and lecturer on his specialty at the University. He has made many original observations embodied in numerous communications to the professional press and learned societies.

Dr. Elizabeth T. Fraser, M.D., one of the Beit Research Fellows, has just drawn attention to a new method of diagnosing pulmonary tuberculosis (*Practitioner*, March 14). This diagnostic measure, which is said to have the advantage of simplicity, is based on the examination of the sputum in suspected cases. For this purpose an emulsion is made by shaking the prepared sputum with chloroform and subsequently filtered. The filtrate is tested in a capillary pipette with an anti-tubercular precipitating serum. When positive, a white precipitating ring appears in the tubes after incubation of about half an hour at 37° C. Dr. Fraser, who has had much experience in bacteriological research, states that the test, although not absolutely conclusive, is a useful aid in doubtful cases.

Drs. W. L. Day, S. Worts, and W. F. A. Clowes, who have been given a dinner at Colchester by the North-East Essex Division of the British Medical Association, received this compliment on account of the hard work they have accomplished in the struggle against the Insurance Act. All three are "Guy's" men, the two latter being in partnership at Colchester. Dr. Day, who is an M.D. Oxon., and Dr. Clowes are colleagues on the staff of the Essex County Hospital.

The new honorary surgeon to the Royal Victoria Infirmary at Newcastle-on-Tyne, Mr. R. J. Willan, is a Durham graduate and obtained first-class honours in the M.S. examination in 1909, having become an F.R.C.S. England in the year previously. He has lately been honorary registrar to the institution where he has now been elected a member of the staff. Among other academic distinctions, Mr. Willan, who is also a demonstrator of surgical pathology at Durham University, obtained the Gibb, Charlton, and Stephen Scott Scholarships. His numerous communications to the medical press include several important observations on Genito-Urinary Surgery.

